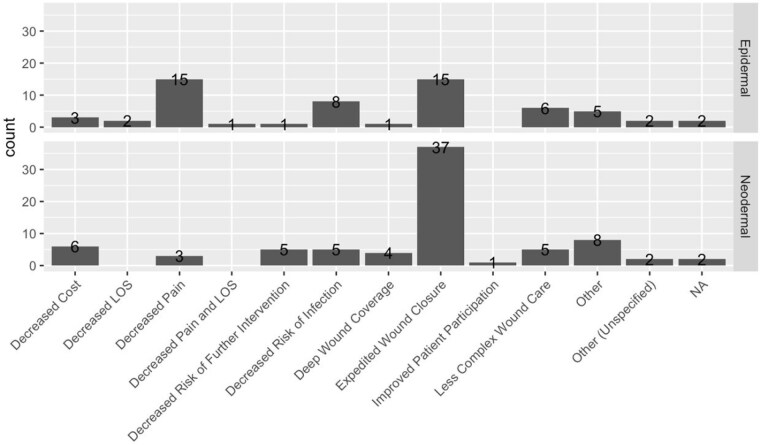# 523 A Survey of Skin Substitute Use Among Burn Surgeons Across the Country

**DOI:** 10.1093/jbcr/iraf019.152

**Published:** 2025-04-01

**Authors:** Deepak Ozhathil, Carter Powell, Joshua Rivers, Henry Ross, Steven Kahn, John Bailey

**Affiliations:** Akron Children’s Hospital; Medical University of South Carolina; Medical University of South Carolina; Medical University of South Carolina; Medical University of South Carolina; Wake Forest University

## Abstract

**Introduction:**

A plethora of “skin substitutes” exist in burn care with limited comparative analysis trials in the literature. This presents a unique challenge to providers seeking to optimize product selection. We sought to perform a cross-sectional survey of practicing burn surgeons to explore what products being utilized and identify the indications for use.

**Methods:**

A 14-question survey was distributed to burn surgeons across the country who attended academic meetings. Contributors were actively practicing burn surgeons and identified the skin substitutes they routinely use in practice and completed a separate product-specific survey on each product, covering aspects such as indication, usage frequency, clinical environment, perceived benefits, and practice changes due to lack of porcine xenograft availability. Statistical analysis involved descriptive statistics, Welch two-sample t-tests, and Pearson’s correlation coefficient.

**Results:**

Contributions were received from 48 surgeons across 39 institutions and 23 US states and Canada in 2022-2023. Over 20 products were reported, On average 4.3 skin substitutes were used per respondent, with no differences between academic and private institutions (p = 0.33) or based on years of practice (r(31) = -0.320, p = 0.069). For neodermal substitutes, a Dermal Regeneration Template (26 respondents) was used for staged full-thickness burns. Polyurethane Foam (PF) (22 respondents), was used similarly and was believed to reduce dressing changes and healing time. Bovine Dermal Scaffold (10 respondents) and Collagen-Elastin Matrix (8 respondents) had mixed feedback. For epidermal substitutes, Polylactic Acid Copolymer (PAC) (20 respondents) was used for middle thickness burns and cited for reduced dressing changes and healing time, whereas Biosynthetic Matrix (BM) (5 respondents) had mixed reviews. Some products were used across multiple indications, including Porcine Lyophilized Extracellular Matrix and Acellularized Human Skin with mixed feedback. Allograft was widely used (28 respondents) for deep partial and full thickness burns. The discontinuation of Porcine xenograft led to increased use of PAC and BM by some respondents. Allograft was preferred for deep partial and mixed thickness burns by most respondents, PF and allograft for full-thickness burns, and PAC for middle thickness burns, cited for expedited wound closure (n=52), decreased pain (n=18) and reduced infection risk (n=13).

**Conclusions:**

Our data highlights the diversity of skin substitute products available to burn surgeons and the lack of consensus on how to utilize these products. The findings highlight the need for more collaboration and consultation between the larger burn community and industry partners to optimize wound indications for product application.

**Applicability of Research to Practice:**

Further research using more comprehensive surveys and blinded outcomes data will help surgeons optimize product selection.

**Funding for the Study:**

N/A